# Pushing the boundaries of behavioral analysis could aid psychiatric drug discovery

**DOI:** 10.1371/journal.pbio.3001904

**Published:** 2022-12-08

**Authors:** Brian M. Sweis, Eric J. Nestler

**Affiliations:** Nash Family Department of Neuroscience, Department of Psychiatry, Friedman Brain Institute, Icahn School of Medicine at Mount Sinai, New York, New York, United States of America

## Abstract

Behavioral changes could be used as a primary phenotypic screen for new drug candidates for psychiatric conditions, if enough useful data can be generated from current behavioral models. In this Perspective, the authors advocate for using machine learning to extract the data we need.

## Teaser

Drug discovery for psychiatric conditions is stagnating. Behavioral changes could be used as a primary phenotypic screen for new drug candidates, if enough useful data can be generated from behavioral models. Could machine learning be the answer to extracting the data we need?

Translational research into mental illness is in dire need of improvement. Treatment options currently available for the vast majority of psychiatric disorders have remained largely unchanged for decades. Most newer medications have the same mechanisms of action as the older ones, and many research efforts have been reduced from “drug discovery” to “drug refinement.” However, the advent of robust tools in molecular, circuit, behavioral, and computational neuroscience, along with recent advancements in machine learning, could offer part of the solution to the problems of stagnation that face psychiatric drug discovery and have only just begun to influence the development of new treatments for psychiatric illnesses.

Drug discovery takes two principal forms: target-based and phenotypic [1]. Target-based drug discovery aims to specifically engage known molecular targets, whereas phenotypic drug discovery relies on interrogating functional biological or behavioral endpoints in a disease-relevant system without necessarily knowing the molecular target of a drug. This is in some ways akin to the origins of psychopharmacology, when drugs were administered to animals and humans without appreciation of a drug’s mechanism of action. Phenotypic drug discovery has seen a general resurgence in the past 20 years, with several first-in-class drugs with no known molecular target recently approved by the US FDA, albeit largely for use outside of psychiatry [1]. We believe that phenotypic drug discovery has the potential to uncover previously unknown disease-relevant cellular signaling pathways or neural circuits and inform a deeper understanding of the pathophysiology of central nervous system diseases. This can be especially useful for psychiatric drug discovery as (i) virtually all psychiatric disorders are still defined today solely by behavioral disturbances and (ii) drug studies in animal models used for psychiatric disorders are limited in behavioral endpoints currently available but are seeing newfound improvement in sophistication with advances in emerging machine learning approaches.

The rate at which drug libraries are being developed is accelerating, with improved compound structural diversity and quality and expanded coverage of physicochemical space [2]. These improvements have been driven in part through cheminformatics, a type of machine learning used to design molecules in silico either based on their known molecular target, on the known actions of a chemically-diverse group of drugs, or completely agnostic to mechanism. Despite this progress, a critical bottleneck remains when promoting drug candidates to in vivo testing. This is especially problematic for behavioral neuroscience, which depends heavily on animal experiments that are labor intensive, generally low throughput, and historically simplistic in nature. Drug candidates to be screened in vivo usually are restricted to, at most, dozens of the many millions of compounds potentially available. Additionally, animal behavioral assays widely used for the study of psychiatric disorders have tried to capture some aspect of a human phenomenon, but traditional approaches are inherently limited in their construct and face validity [3]. For example, the tail suspension and forced swim tests have served for many years as rodent “models of despair” or “learned helplessness” that, while initially useful for their ease of screening, are now rightly being met with increasing scrutiny and falling out of favor.

**Fig 1:**
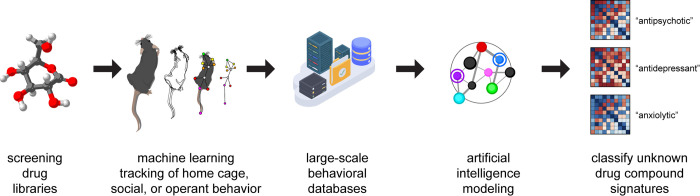
Behavior-based phenotypic drug discovery driven by machine learning. Illustration of a generic pipeline for behavior-based phenotypic drug discovery. Large libraries of drug compounds are developed and screened in silico before being selected for in vivo behavioral testing. Advances in machine learning approaches to animal tracking, either naturalistic behavior in the home cage or more sophisticated measurements taken during task engagement, are capable of extracting increasingly complex behavioral features. Machine learning modeling of large-scale behavioral datasets derived from experiments probing the behavioral effects of experimental drug compounds can generate phenotypic signature profiles revealing previously unknown drug properties in an unsupervised manner.

Within this context, we suggest that advances in machine learning should be used to jumpstart drug discovery efforts in psychiatry by taking greater advantage of the richness of animal behavior in two ways. The first is to use behavior as a primary screen of compounds by achieving much higher throughput with automated, machine learning–based approaches. Some platforms, such as SmartCube, aim to increase automation in order to detect spontaneous and evoked behavioral profiles either in a novel environment or home cage [4]. These approaches train classification algorithms to map complex behavioral features on to a reference database built from dose–response curves of hundreds of known drugs based on their class, mechanism of action, or clinical indication. In this way, novel compounds can be screened en masse (Fig 1). Other machine learning approaches, such as DeepLabCut, are aimed at improving the sophistication of animal behavior tracking and include robust, open-ended, automated systems that can characterize large-scale, unbiased home cage behaviors in healthy and diseased states [5,6]. These innovative approaches are distinct from most current efforts to automate rodent behavioral analyses—which seek mainly to reduce imprecision and variability in human observation—and we expect such approaches to enhance phenotypic drug discovery by “automating serendipity.” Indeed, the open-ended use of behavioral endpoints as a primary drug screen has already led to the development of several compounds currently in clinical trials, most of which have no known molecular target despite producing behavioral profiles similar to existing drug classes [7].

The second way to take advantage of animal behavior studies is to use machine learning to extract more insight from established, but highly complex, behavioral procedures—an area of study that we think is especially fertile for rich exploration and holds great promise for the future of behavioral neuroscience [8]. For example, an “addiction index” composite score made up of numerous task-dependent behaviors involved in drug self-administration by rodents may more accurately capture disease-relevant pathology than single measures (e.g., drug intake or lever pressing) [9]. Another example is the neuroeconomic task, “Restaurant Row,” in which rodents forage for their sole source of food by making serial decisions to accept or reject offers of varying delays and flavors while on a limited time budget [10]. This task forces individuals to make economic choices that inherently depend on energy optimization but also on subjective preferences. Restaurant Row thereby operationalizes reward value across numerous behavioral dimensions that depend on different decision-making strategies, can tap into different neural circuits, and, we think, can help push boundaries on what behaviors can be studied in animals for psychiatric research [11]. This task can be used in rodents, monkeys, and humans and has been adapted to match ethology across evolution and emphasizes improving a paradigm’s computational validity, that is, translating not only behavior but also the brain’s algorithms across species [12]. The vast amounts of data that result from such behavioral datasets could then be leveraged to better understand aspects of cognition and emotional responses under healthy and pathological conditions. Although we view these approaches as still being hampered by limited throughput in terms of drug screening, such open-ended, machine learning–based analyses of complex behavior are being guided across species, including humans, which promises a higher degree of validity for the animal studies and more effective translation into the clinic [6,12].

Finally, machine learning is advancing efforts to understand the strategies and circuit mechanisms by which the brain processes (computes) information and generates and executes an action. As all psychiatric illnesses are currently categorized solely by behavioral abnormalities that are inherently unknowable in animals (e.g., hallucinations, delusions, or abnormal mood), we think that explicating neural networks that underlie complex symptoms in humans will ultimately lead to more effective and personalized therapeutics [13].

These are early days for the application of machine learning to psychiatric drug discovery, but we are certain that these powerful tools can better equip scientists and clinicians to gain a deeper understanding of healthy and diseased brain function with the promise of propelling the field of psychiatry into the modern era. As physician-scientists working across basic neuroscience and clinical psychiatry practice, we encourage more groups to move beyond simple tests of animal behavior and urge greater consideration of ways to innovate and translate behavioral discoveries across species in order to ultimately improve what we can do for patients living with mental illnesses.
